# Variations in Soil Functional Fungal Community Structure Associated With Pure and Mixed Plantations in Typical Temperate Forests of China

**DOI:** 10.3389/fmicb.2019.01636

**Published:** 2019-07-16

**Authors:** Di Wu, Mengmeng Zhang, Mu Peng, Xin Sui, Wei Li, Guangyu Sun

**Affiliations:** ^1^College of Life Science, Northeast Forestry University, Harbin, China; ^2^Heilongjiang Provincial Key Laboratory of Ecological Restoration and Resource Utilization for Cold Region, School of Life Sciences, Heilongjiang University, Harbin, China; ^3^College of Resources and Environmental Science, Northeast Agricultural University, Harbin, China

**Keywords:** forest ecosystems, pure and mixed plantations, ectomycorrhizal fungi, saprotrophic fungi, high-throughput sequencing

## Abstract

Forest plants are in constant contact with the soil fungal community, which plays an important role in the circulation of nutrients through forest ecosystems. The objective of this study was to evaluate the fungal diversity in soil and elucidate the ecological role of functional fungal communities in forest ecosystems using soil samples from seven different plantations in northeastern China. Our results showed that the fungal communities were dominated by the phyla Ascomycota, Basidiomycota, and Mortierellomycota, and the mixed plantation of *Fraxinus mandshurica* and *Pinus koraiensis* had a soil fungal population clearly divergent from those in the other plantations. Additionally, the mixed plantation of *F. mandshurica* and *P. koraiensis*, which was low in soil nutrients, contained a highly diverse and abundant population of ectomycorrhizal fungi, whereas saprophytic fungi were more abundant in plantations with high soil nutrients. Redundancy analysis demonstrated a strong correlation between saprophytic fungi and the level of soil nutrients, whereas ectomycorrhizal fungi were mainly distributed in soils with low nutrient. Our findings provide insights into the importance of functional fungi and the mediation of soil nutrients in mixed plantations and reveal the effect of biodiversity on temperate forests.

## Introduction

Soil fungi play a crucial role in the ecosystem diversity and functional reconstruction and represent an essential functional component of soil and ecological systems as decomposers, symbionts, and pathogens. Furthermore, fungi are closely involved in the energy flow, nutrient cycle, and transformation of organic substances in soil (Cromack et al., 1977; Buee et al., 2009; Li et al., 2018). Their biomass and structure could provide an early indication of changes in soil characteristics, thereby making fungi more credible than organic matter as indicators of environmental changes (Powlson et al., 1987; Blagodatskaya and Kuzyakov, 2008). Spatial and temporal variations in soil microorganisms are affected by differences in dominant tree species, which can ultimately alter the availability and dynamics of soil nutrients, and cause shifts in the composition of microbial communities as they adapt to new environmental conditions (Priha and Smolander, 1997; White et al., 2005; Saetre and Bååth, 2009; Nagati et al., 2018).

Ectomycorrhizal (ECM) and saprophytic fungi are important as two major fungal guilds in forest ecosystems, especially in temperate regions (Liu Y.P. et al., 2018). ECM fungi play a determinant role in the absorption of nutrients and water by plants through establishing mutualistic interactions with plant roots (Becquer et al., 2018; Clasen et al., 2018). Saprophytic fungi are mainly responsible for the decomposition of complex organic matter and conversion of nutrients (Talbot et al., 2013; Nagati et al., 2018). Plant diversity can greatly affect the composition of saprotrophic fungi and increase their community diversity by providing a wide variety of substrates to establish facilitative interactions with them (Gessner et al., 2010; Zhang et al., 2018).

Forest plantation or reforestation represents a significant silviculture and forest management practice worldwide (Evans, 1992; Paquette and Messier, 2010); however, the establishment of large-scale and continuous pure plantations can cause many problems, including the biodiversity loss, soil degradation, and a decline in ecosystem stability. To improve the ecological functions and economic value of reforestation, a planting model of a mixed forest, with various different tree species, has been gradually adopted when establishing new plantations. Recently, many studies have described the most important afforestation tree species, based on their effects on microbial biomass and community structure (Chen et al., 2005; Busse et al., 2006; Wang W.X. et al., 2013).

The effects of pure and mixed plantations on microbial communities have been reported; however, there are relatively few studies on functional soil fungal communities in forest plantations (Ushio et al., 2010; Flores-Rentería et al., 2016; Žifčáková et al., 2016). *Juglans mandshurica* and *Fraxinus mandshurica* are important hardwood broad-leaved tree species in Northeast China (Sun and Liu, 2015). *Pinus koraiensis* is a constructive tree species of the top vegetation community during natural succession in northeastern China; it is an important component of coniferous-broad leaf mixed forests in the cold temperate zone, and its dynamic change is related to the stability of forest ecosystems in northeast China (Liu et al., 2016; Wang et al., 2019). In this study, we determined the fungal diversity and characteristics of functional fungal communities in soil under three important afforestation tree species (*P*. *koraiensis*, *J. mandshurica*, and *F. mandshurica*) in northeastern China, as well as the relative roles of ECM and saprotrophic fungal communities in pure and mixed forests. These results provide a better understanding of the ecological functions of the soil–fungal community between mixed and pure plantations, and reveal preliminarily underlying mechanisms between soil nutrients and soil–fungal functional community, especially for those tree species used in forest plantation.

## Materials and Methods

### Study Site and Sampling

The experimental site was located in the Maoershan Forestry Experimental Station of Northeast Forestry University, Heilongjiang Province, China, at a latitude of 45°21′–45°25′N, a longitude of 127°30′–127°34′E, and an altitude of 390 m. The area is characterized by a temperate continental monsoon climate with the mean annual precipitation of 723 mm and the mean annual air temperature of 2.8°C. The zonal soil is a dark brown earth (Gu et al., 2010).

The test stands were reforested with pure or mixed plantations in 1986. Five different plantations were selected, including (1) pure *P. koraiensis* forest (PK), (2) pure *J. mandshurica* forest (JM), (3) pure *F. mandshurica* forest (FM), (4) mixed forest of *P. koraiensis* and *J. mandshurica* (P × J), and (5) mixed forest of *P. koraiensis* and *F. mandshurica* (P × F). The coniferous forest (PK), broad-leaved forests (JM and FM), and mixed coniferous and broad-leaved forests (P × J and P × F) were established at the same site by strips (each pure or mixed forest of ∼0.5 ha), with a planting row space of 2 m × 1.5 m. Each plantation was separated by an interval zone.

Soil samples were collected in July 2014 from the soil in the root zone under the different forests. In the two mixed plantations, samples were collected from the soil under each tree species. Therefore, there were seven treatments, namely, PK, JM, FM, *J. mandshurica* from P × J [JM(P × J)], *P. koraiensis* from P × J [PK(P × J)], *F. mandshurica* from P × F [FM(P × F)], and *P. koraiensis* from P × F [PK(P × F)]. Three plots (10 m × 20 m each) were set per treatment, and the soil samples from each plot were collected at a depth of 0–10 cm using a 10-spot sampling method and mixed. We removed impurities such as rocks, plant roots, and other objects. Chemical properties were determined for all soil samples, and the samples were stored at -80°C until DNA extraction for fungal community analysis.

### Determination of Soil Chemical Properties

To analyze its chemical properties, soil was air-dried at room temperature and subsequently sieved. Total carbon (TC) content was determined using a total organic carbon analyzer (Vario, Elementar, Langenselbold, Germany). Total nitrogen (TN) content was determined using a Kjeldahl apparatus (BUCHI, Ltd., Flawil, Switzerland). Total phosphorus (TP), available phosphorus (AP), and alkali-hydrolyzable nitrogen (AHN) content were determined as reported previously (Olsen and Sommers, 1982; Lu, 2000; Khan et al., 2001). Soil pH was measured in a 1:2.5 soil/water suspension using a pH meter (PHS-3C; INESA Scientific Instrument Co., Ltd., Shanghai, China) (Dong et al., 2014). The potential nitrification (PN) rates were measured according to ISO 15685 (2012). Graphs were generated using the SigmaPlot software (v.11.0; Systat Software, Inc., San Jose, CA, United States).

### High-Throughput Sequencing of Internal Transcribed Spacer (ITS) Regions

Genomic DNA was extracted from 250 mg of fresh soil using a PowerSoil DNA isolation kit (Mobio Laboratories, Inc., Carlsbad, CA, United States) according to the manufacturer instructions. Polymerase chain reaction (PCR) amplification was conducted using the primer set ITS1F (5′-CTTGGTCATTTAGAGGAAGTAA-3′) and ITS2R (5′-GCTGCGTTCTTCATCGATGC-3′) (Adams et al., 2013). Each primer contained a barcode unique to each sample. The PCR conditions included initial denaturation at 95°C for 5 min, followed by 29 cycles of 95°C for 30 s, 55°C for 30 s, 72°C for 45 s, and a final extension at 72°C for 5 min. Amplicon sequencing was performed on the Illumina HiSeq platform according to standard protocols.

### Processing of ITS Sequencing Data

Data generated by sequencing were processed and analyzed using the QIIME pipeline (version 1.8.0) (Caporaso et al., 2010). Forward and reverse reads were merged using Flash (version 1.2.11). Sequences shorter than 200 bp were truncated with a quality score <20 over a 50-bp sliding window (Xu et al., 2017). Exact barcode matching was used, allowing a two-nucleotide mismatch. Reads containing ambiguous bases were removed. Only sequences that overlapped by >10 bp were assembled. Chimeras were screened and removed using the UCHIME algorithm. Sequences were clustered into operational taxonomic units (OTUs) at a 97% identity threshold using the UPARSE software package in the Usearch platform (version 7.1^1^). A representative sequence for each OTU was assigned to annotate taxonomic information using the UNITE community database. To equalize read sizes for their comparison among soil samples, random subsampling was conducted with the lowest value (50,328 reads) for further standardization analysis. Rarefaction analysis was performed for each sample. The Mothur software (version v.1.35.0) was used to estimate coverage according to Good’s estimator; community richness and diversity were estimated, using the Chao index, abundance coverage estimator (ACE), and Shannon index. The H-cluster of each sample was analyzed at the OTU level using the R software’s Vegan package based on the Bray Curtis dissimilarity distance matrix and pairwise comparison of each sample and assessed by multivariate permutational analysis of variance (PERMANOVA). The R software (Venn diagram package) was used to depict a Venn diagram. The obtained OTUs were assigned into fungal functional guilds using the FUNGuild database (Nguyen et al., 2016).

### Statistical Analyses

Differences in soil chemical properties, read numbers of dominant OTUs, and α-diversity indices (all fungi, ECM, and saprotrophic fungi) were assessed using ANOVA in SPSS 19.0 (SPSS Inc., Chicago, IL, United States), and a *p*-value of 0.05 was considered statistically significant (Shen et al., 2016). Redundancy analysis (RDA) was performed using the CANOCO software (Canoco for Windows 4.5, Microcomputer Power Inc., Willis, TX, United States) to test the relationships among genera and soil chemical properties according to the method described by Van Den Wollenberg (1977). Statistical significance of the relative abundance data at the phylum and class levels was analyzed using the STAMP software (Parks et al., 2014). Co-occurrence network analysis was performed using relative abundance values of OTUs with a Spearman correlation coefficient (*r*) >0.7 and *p*< 0.05, and the data were visualized using Cytoscape (version 3.4.0), according to a previous study (Shannon et al., 2003).

### Data Access

All the fungal raw sequences have been deposited to GenBank Short Read Archive (No. SRP191732).

## Results

### Soil Chemical Properties

The properties of the different soils are shown in Table 1. TN and TC contents, pH, and the PN rates of the soil in the mixed plantation of *P.* *koraiensis* and *J. mandshurica* were much higher than that those in the corresponding pure plantations (*p <*0.05); and although not significantly different, the soil AP levels were also higher in P × J than in the pure plantations. Conversely, TN and TC contents and the PN rates of the soil in the mixed plantation of *P.* *koraiensis* and *F*. *mandshurica* were lower than those in the corresponding pure plantations, with some values showing significant differences (*p* < 0.05). Soil TP content, AHN level, and C/N ratio mainly showed downward trends in both mixed plantations, although no significant differences were found. Generally, the levels of soil nutrients in the mixed plantation of *P.* *koraiensis* and *J. mandshurica* were higher than those in the mixed plantation of *P.* *koraiensis* and *F*. *mandshurica*. These results indicated that the mixture of different plant species could alter soil nutrient levels.

**Table 1:** Soil chemical properties in seven different plantation types.

Type	pH	Total nitrogen	Total phosphorus	Total carbon	C/N ratio	Alkaline hydrolyzable nitrogen	Available phosphorus	Potential nitrification rate
		
		(mg g^-1^)	(mg g^-1^)	(mg g^-1^)		(mg kg^-1^)	(mg kg^-1^)	(μg NO_2_- N g^-1^ h^-1^)
PK	5.09 ± 0.07a	4.48 ± 0.52ab	1.01 ± 0.04abc	84.0 ± 1.7b	18.92 ± 1.97a	369.7 ± 48.4a	21.0 ± 0.7a	0.93 ± 0.10d
JM	5.46 ± 0.05cd	4.44 ± 0.72ab	1.13 ± 0.04c	110.1 ± 9.9c	25.01 ± 2.35b	348.6 ± 103.9a	19.4 ± 1.9a	0.84 ± 0.03c
JM(P × J)	5.51 ± 0.06d	6.88 ± 0.36d	1.04 ± 0.13bc	136.1 ± 1.6d	19.82 ± 1.27a	412.9 ± 52.5a	29.6 ± 0.7b	1.93 ± 0.03f
PK(P × J)	5.42 ± 0.03c	5.55 ± 0.27c	0.98 ± 0.03ab	105.2 ± 1.4c	18.97 ± 1.09a	309.9 ± 68.9a	22.4 ± 8.1a	1.71 ± 0.04e
FM	5.40 ± 0.05c	4.75 ± 0.05b	1.03 ± 0.08bc	85.8 ± 3.1b	18.06 ± 0.57a	383.7 ± 21.9a	21.7 ± 2.7a	0.93 ± 0.03d
FM(P × F)	5.27 ± 0.02b	3.89 ± 0.14a	0.96 ± 0.03ab	80.8 ± 3.7b	20.78 ± 1.08a	284.8 ± 13.2a	25.0 ± 3.5ab	0.27 ± 0.02a
PK(P × F)	5.22 ± 0.04b	3.82 ± 0.06a	0.89 ± 0.01a	71.5 ± 2.8a	18.73 ± 0.49a	290.6 ± 17.1a	19.2 ± 2.3a	0.58 ± 0.01b
*F*	33.270	23.684	3.887	75.883	8.362	2.356	2.945	529.129
*P*	<0.001	<0.001	0.017	<0.001	0.001	0.088	0.045	<0.001


*The different letters indicate a significant difference among the seven plantations, Duncan’s multiple range test. TC, total carbon; TN, total nitrogen; PN, the potential nitrification rate; TP, total phosphorus; AP, available phosphorus; AHN, alkaline hydrolyzable nitrogen.*

### Fungal Diversity and Richness

The number of OTUs increased with the number of reads in each sample, and the number of ITS sequences reached a saturation plateau in all soil types, indicating that new fungal phylotypes would not be detected with the increase in the number of reads (Supplementary Figure S1). Good’s coverage estimator for all plantation types was >99%, demonstrating that the number of sequences was adequate to reveal the fungal diversity in different forest types.

The richness indices (ACE and Chao) for the mixed forest plantation types, except for the FM(P × F) sample, showed increasing trends compared with those for the corresponding pure forests, although the differences were not statistically significant (Table 2). Additionally, the OTU numbers and fungal diversity (Shannon index) in the soil varied among the different plantation types, showing increases in the mixed plantation of *P.* *koraiensis* and *J. mandshurica* and decrease in the mixed plantations of *P.* *koraiensis* and *F*. *mandshurica* relative to the values obtained for the corresponding pure plantations, with no significant differences.

**Table 2:** Richness and diversity estimators of soil–fungal community in the seven different types of plantation.

Sample	OTUs		ACE		Chao		Shannon	
	Mean	*SE*	Mean	*SE*	Mean	*SE*	Mean	*SE*
PK	775	46.5	806	38.3	813	43.0	4.57	0.46
JM	890	76.2	931	59.4	937	59.5	4.77	0.35
JM(P × J)	927	98.1	955	86.3	969	84.2	4.99	0.52
PK(P × J)	869	135.2	911	119.6	926	112.1	4.48	0.79
FM	804	114.5	843	73.0	846	75.3	4.47	1.04
FM(P × F)	725	130.7	810	130.7	818	138.5	3.89	0.80
PK(P × F)	755	159.5	833	147.5	840	156.4	4.39	0.60
*F*	1.149		1.101		1.145		0.751	
*P*	0.385		0.409		0.387		0.619	


*SE, standard error.*

### Fungal Taxonomic Differences Between Mixed and Pure Forests

**FIGURE 1:**
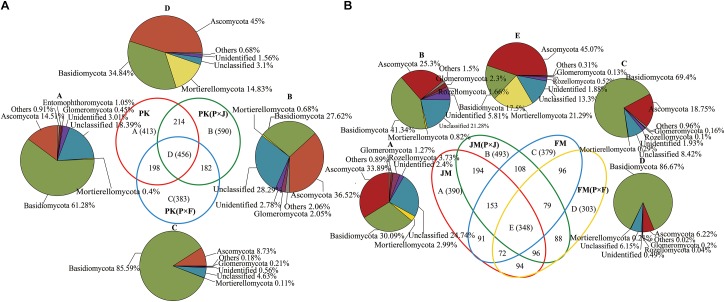
Venn diagram showing the number of unique and shared species in soil samples from pure and mixed plantations of **(A)** *P.* *koraiensis* and **(B)** *J. mandshurica* or *F*. *mandshurica*.

We constructed a Venn diagram to analyze the common and unique fungal OTUs among pure and mixed plantations (Figure 1). Figure 1A shows that 456 OTUs were common among the pure and mixed plantation types of *P.* *koraiensis*, with the majority of OTUs assigned to Basidiomycota (34.84%), Ascomycota (45.00%), and Mortierellomycota (14.83%). Additionally, 590 and 383 unique fungal OTUs were found in the PK(P × J) and PK(P × F) samples, respectively, with the number of fungal OTUs in the PK(P × J) soil samples much higher than that in the PK(P × F) soil samples. Similar results were obtained by analyzing soil samples from the pure and mixed broad-leaved plantations, including JM, JM(P × J), FM, and FM(P × F), with the majority of the 348 shared OTUs assigned to Basidiomycota (17.5%), Ascomycota (45.07%) and Mortierellomycota (21.29%) (Figure 1B). Moreover, the number of unique fungal species (493) was higher in JM(P × J) than in the other types of forest. These results indicated that the number of unique fungal OTUs in the soil fungal community was higher in the mixed plantation of *J. mandshurica* and *P. koraiensis* than in the pure plantation of either species, whereas the opposite trend was observed in the mixed plantations of *P.* *koraiensis* and *F*. *mandshurica*.

### Fungal Community Composition

**FIGURE 2:**
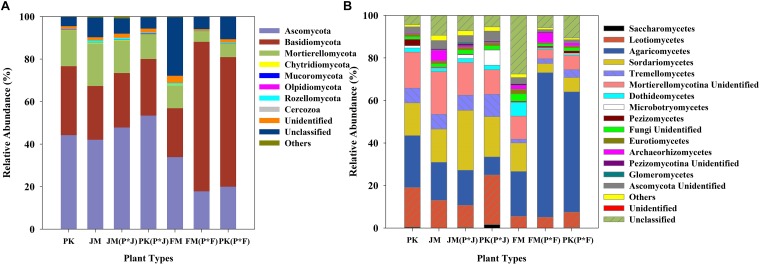
Relative abundances of **(A)** fungal phyla and **(B)** classes in each plantation.

The identified sequences from the different plantation types were affiliated with 15 fungal phyla (Figure 2A), among which Ascomycota, Basidiomycota, and Mortierellomycota were predominant in the soils. These dominant fungal phyla were found in all soil samples, and comparative analysis revealed a distinct distribution of the predominant fungi. The mixed plantation of *P.* *koraiensis* and *F*. *mandshurica* was characterized by a significantly higher abundance of Basidiomycota and significantly lower abundance of Ascomycota, whereas the other pure and mixed forest soils showed a lower abundance of Basidiomycota and higher abundance of Ascomycota (Supplementary Figure S2).

At the class level, 11 dominant classes were identified, which are shown in Figure 2B. The fungal communities had a similar taxonomic distribution at the class level across all plantation types, and were dominated by Agaricomycetes, Leotiomycetes, Sordariomycetes, and Tremellomycetes. Sordariomycetes, Tremellomycetes, and Leotiomycetes were predominant in the mixed plantation of *P.* *koraiensis* and *J. mandshurica*, with a much higher abundance than that in the mixed plantation of *P.* *koraiensis* and *F*. *mandshurica*. However, Agaricomycetes showed a significantly higher abundance in the mixed plantation of *P.* *koraiensis* and *F*. *mandshurica* than in most of the other forest types (*p*< 0.01 or *p*< 0.001) (Supplementary Figure S3).

The dominant fungal OTUs are shown in Supplementary Table S1. Most of the dominant fungal OTUs reached significant levels in the different plantations. The numbers of reads for OTU1432, OTU2963, and OTU3597 were significantly higher in the pure plantations of *P.* *koraiensis*, *J. mandshurica*, and *F*. *mandshurica*, and in the mixed plantation of *P.* *koraiensis* and *J. mandshurica*, whereas there was a significantly greater number of reads for OTU1421 in the mixed plantation of *P.* *koraiensis* and *F*. *mandshurica*.

We then performed cluster analysis based on the relative abundance of OTUs in soils from the different plantations (Supplementary Figure S4). The results revealed separate clusters for the PK(P × F) and FM(P × F) samples and JM(P × J), JM, PK, FM, and PK(P × J) samples. The PERMANOVA analysis indicated significant differences between the two clusters (*r*^2^ = 0.25, *p*= 0.001), suggesting differences in the fungal communities between the two groups.

**FIGURE 3:**
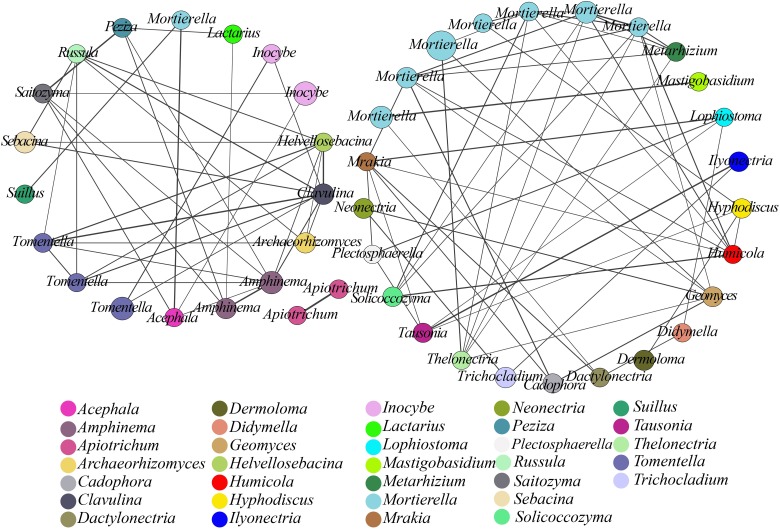
Network analysis showing fungal co-occurrence patterns in soil samples from different plantations. All OTUs with an average abundance >0.2% in all samples were included. The size of each node is proportional to the relative abundance of the fungal OTUs, and nodes in the network are marked with identical colors for the same genera. The thickness of each solid line is proportional to the *r*-value.

We used co-occurrence analysis to evaluate the relationships among the microbial communities at the species level (Xiao et al., 2016). A co-occurring network of these dominant fungal OTUs was plotted to show positive relationships, with 45 OTUs (nodes) displaying positive associations based on the correlation analysis (Figure 3) and connections (91 lines) in the network representing strong (*r* > 0.7) and significant (*p*< 0.05) correlations. These dominant OTUs in the network belonged to 33 genera, with *Mortierella* being the most abundant. This finding suggested a considerable connection of *Mortierella* with other OTUs or genera based on the presence of 32 edges and high *r*-values.

### Functional Fungal Populations

**FIGURE 4:**
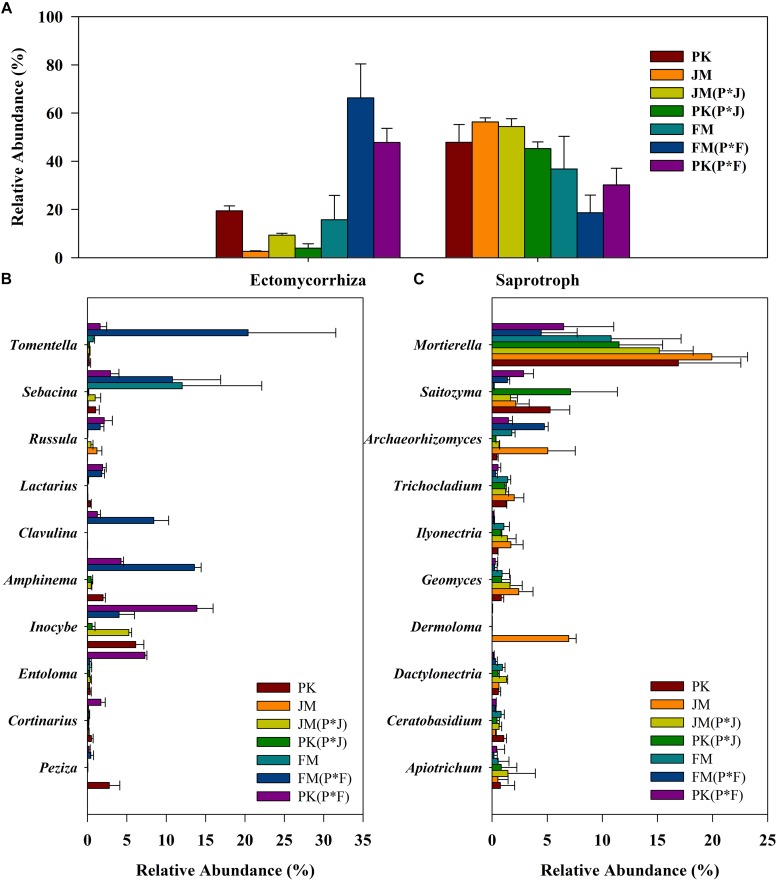
Relative abundance of **(A)** the total ECM and saprotrophic fungi, **(B)** dominant ECM fungal genera, and **(C)** dominant saprotrophic fungal genera in different plantations.

Most of the fungi were mainly divided into ECM and saprotrophic taxa (including the ECM–saprotroph type). The ECM and saprotrophic fungal communities were compared among the different plantation soils according to their total abundance (Figure 4A) and α-diversity indices (Supplementary Tables S2, S3). The total abundance of ECM fungi was significantly higher in the soil under the mixed plantation of *P.* *koraiensis* and *F*. *mandshurica* than in that under the other plantations. Additionally, saprotrophic fungi showed significantly higher total abundance in the soils under the mixed and pure plantations of *P.* *koraiensis* and *J. mandshurica* relative to that in the mixed plantation of *P.* *koraiensis* and *F*. *mandshurica* (Supplementary Figure S5). A similar pattern was observed for the α-diversity of saprotrophic fungi, whose greater richness and diversity indices (ACE and Chao) in the mixed plantation of *P.* *koraiensis* and *J. mandshurica* [PK(P × J) and JM(P × J)] significantly differed from those of other samples. Meanwhile, the ECM fungi from the mixed plantation of *P.* *koraiensis* and *F*. *mandshurica* showed greater richness and diversity indices relative to those in the other plantations (Supplementary Tables S2, S3).

Regarding functional genus-based analysis, 52 and 290 identified genera were assigned to ECM and saprotrophic genera, respectively; however, the relative proportions of the ECM and saprotrophic genera differed among the different plantation types (Figure 4B,C). Although the biodiversity was quite stable, changes in the relative abundance of some specific genera were obvious. The most abundant saprotrophic genus was *Mortierella*, whereas *Tomentella*, *Sebacina*, and *Inocybe* were predominant ECM genera, according to their total abundance, especially in the mixed plantation of *P.* *koraiensis* and *F*. *mandshurica*.

Overall, the results clearly showed significantly higher proportions of ECM taxa and lower proportions of saprotrophic taxa in the mixed plantation of *P.* *koraiensis* and *F*. *mandshurica*, whereas no significant differences in the total abundance of ECM and saprotrophic taxa were observed in the mixed plantation of *P.* *koraiensis* and *J. mandshurica* relative to those in the pure plantations.

### Relationships Between Soil Chemical Properties and Functional Fungal Genera

**FIGURE 5:**
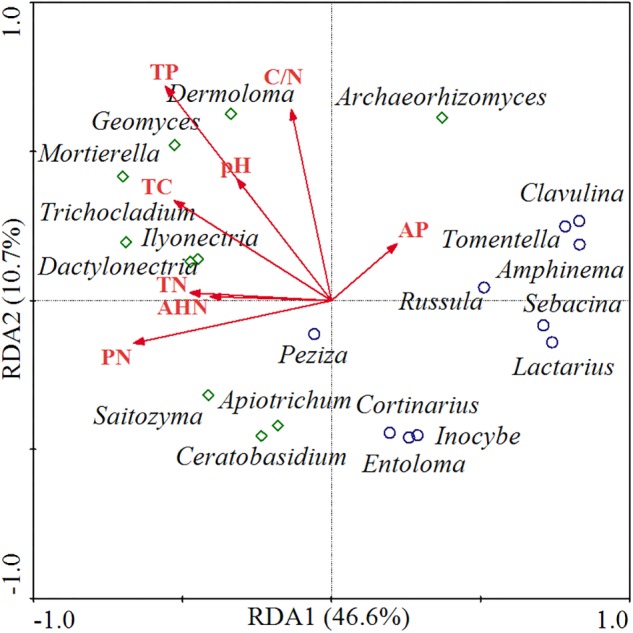
RDA of functional fungal communities and soil chemical properties. TC, total carbon; TN, total nitrogen; PN, potential nitrification rate; TP, total phosphorus; AP, available phosphorus; AHN, alkali-hydrolyzable nitrogen. Red lines represent the soil chemical parameters, green diamonds represent the saprotrophic fungal genera, and blue circles represent the ECM fungal genera.

The relationships between functional fungal communities and soil chemical properties were visualized through RDA ordination (Figure 5). The first two principal components explained 46.6 and 10.7% of variance, respectively, and the ordination diagram shows a distinct distribution between saprotrophic and ECM fungal communities and soil nutrients along the RDA1 axis. Thus, saprotrophic fungi were positively correlated with most of the soil nutrient factors, which indicated that the relative abundance of saprotrophic fungi was higher when soil was nutrients rich. Meanwhile, ECM fungi were negatively correlated with most of the soil nutrient factors, implying that a soil low in nutrients might induce an increase in the abundance of ECM fungi. Furthermore, saprotrophic fungi were mainly distributed in higher-pH areas, whereas ECM fungi were mainly distributed in lower-pH areas.

## Discussion

Microorganisms play a key role in forest soil ecosystems, and maintain an ecosystem balance in forest soils through decomposition of soil organic matter and processes of nutrient mineralization (Comerford et al., 2013; Richter et al., 2018). Additionally, microorganisms present in soil directly or indirectly influence soil nutrient transformations (Ingham et al., 1985; Van Der Heijden et al., 2008). Previous studies have demonstrated that pure forest plantations or long-term continuous cropping systems decrease soil nutrient consumption and fertility, thereby increasing nutrient imbalances or decreasing soil–microbial activity and diversity by altering the population structure (Reeves, 1997; Wang H. et al., 2013; Xiong et al., 2015). In the present study, we found that variations in the soil nutrient content and functional fungal communities under the mixed plantation of *P. koraiensis* and two broad-leaved tree species were different from those observed in pure-forest stands.

Most forests are multi-species and heterogeneous (mixed forests). The positive effects of mixing species have been widely recognized in silviculture, especially for certain tree species with different functional traits (Cannell et al., 1992). Compared with monocultures, mixed forests may exhibit greater levels of ecosystem functions and services, including enhanced productivity, improved soil nutrients, and increased soil microbial diversity and enzyme activity (Singh et al., 2012; Seidel et al., 2013; Forrester, 2014). A previous study has indicated that soil microorganisms in mixed forests are better than those in pure forests (Jiang et al., 2012). In the present study, richness indices (ACE and Chao) were higher in soil samples from mixed plantations than in those from corresponding pure forests, with the exception of the FM(P × F) sample. Weber et al. (2014) proposed that the elevation in richness tends to weaken the dominance of the most competitive species in an ecosystem and promote the harmonious coexistence of microbial taxa. Similarly, Chauvat et al. (2011) demonstrated that the biodiversity of the soil faunal community could increase during conversion from pure stands to mixed forest stands. These findings suggest that establishing mixed plantations not only changes soil nutrients but also directly affects soil fungal community and may provide more fungus-mediated benefits to plant and soil ecosystems.

We found that 456 common OTUs were shared among soils from pure and mixed plantations of *P.* *koraiensis* (Figure 1A), with the dominant phyla mainly being Ascomycota, Basidiomycota, and Mortierellomycota. Similarly, 348 common OTUs from soils under the pure and mixed plantations of *J. mandshurica* and *F*. *mandshurica* were also primarily assigned to Ascomycota, Basidiomycota, and Mortierellomycota (Figure 1B). These common taxa can be deemed as the core microorganisms in forest soils and defined as the group of members shared among microbial communities (Turnbaugh et al., 2007; Xiao et al., 2016). Moreover, these core microorganisms may play vital roles in the function and stability of the microbiota (Xiao et al., 2016). Shade and Handelsman (2012) proposed that identifying core species or OTUs is essential for elucidating the ecology of microbial consortia and that microorganisms associated with a particular habitat are likely critical for the community function. Furthermore, we found that the number of unique OTUs was significantly higher in the mixed plantation of *P.* *koraiensis* and *J. mandshurica* than in the corresponding pure plantations. By contrast, the number of unique OTUs was lower in the mixed forest of *P. koraiensis* and *F. mandshurica* than in the corresponding pure plantations. These findings suggest differences in the interactions between *P. koraiensis* and other broad-leaf tree species, depending on the biological and chemical properties of the soil. Identification of the species represented by unique OTUs may better reflect changes introduced by different planting practices (Ma et al., 2016). Additionally, the proportion of unique OTUs representing unclassified fungi was higher in samples from the mixed forest of *P.* *koraiensis* and *J. mandshurica*. It is possible that some unknown functional fungi are represented by unclassified OTUs. In our future work, we will focus on these unknown species to elucidate their ecological functions.

In the present study, we found statistically significant differences in the fungal community structure regarding the dominant classes (Supplementary Figure S3). The abundance of the dominant classes distinctly varied among the different forest types, with Leotiomycetes, Tremellomycetes, and Sordariomycetes showing significant abundance in the mixed forest of *P.* *koraiensis* and *J. mandshurica*. The class Leotiomycetes comprises a variety of fungi with different ecological functions, which contribute to wood and plant litter decay (Boberg et al., 2011). Sordariomycetes represent well-characterized cellulolytic taxa of major degraders of cellulose (Wilhelm et al., 2017), and members of Tremellomycetes have a high potential for wood rot activity as saprotrophs (Millanes et al., 2011; Gusman et al., 2014). Members of cellulolytic taxonomic groups, as decomposers of plant litter, can critically influence soil nutrient cycling, and physicochemical characteristics (Song et al., 2015; Wilhelm et al., 2017). Notably, the only functional class displaying significant dominance in soil samples from the mixed forest of *P. koraiensis* and *F. mandshurica* was Agaricomycetes, representing ECM fungi, whereas the relative abundance of other functional classes was reduced, likely due to low soil nutrient levels. Low soil nutrients also explain the reduced diversity of functional fungal classes in the mixed plantation of *P. koraiensis* and *F. mandshurica*, which may indicate that Agaricomycetes are strong competitors under these conditions.

Fungal communities were further analyzed based on functional guilds to better understand their ecological specificity (Detheridge et al., 2018). Community differences among different plantations were largely attributable to differences in the relative abundance of major functional fungi. In this study, we focused on ECM and saprotrophic taxa and observed higher ECM fungal diversity and abundance in the mixed plantation of *P.* *koraiensis* and *F*. *mandshurica*, with lower soil nutrients, which may indicate the existence of a mechanism enabling the regulation of soil low in nutrients. ECM fungi can excrete extracellular enzymes that degrade complex organic nitrogen compounds, thereby providing benefits to forest trees by enhancing the mobilization and uptake of soil nutrients (Nygren et al., 2007; Courty et al., 2010; Li et al., 2016; Luo et al., 2018). Additionally, ECM symbiosis is usually viewed as promoting nutritional mutualism and providing resources for host trees to withstand harsh conditions (Peay, 2016; Karst et al., 2018). Previous studies have shown that plants likely form different root symbioses via mycorrhizal networks, such as those associated with ECM fungi, to promote the growth of their neighbors (Simard et al., 2012; Teste et al., 2014; Luo et al., 2018). Therefore, if a mycorrhiza-mediated network mechanism does exist in forests, it may not only facilitate the assimilation of soil nutrients by mixed plant communities under poor nutrient conditions (Luo et al., 2018) but also greatly promote the co-existence of plant species during reforestation, especially in the cases of mixed ECM and non-ECM plant species.

Although we observed relatively similar levels of diversity and richness among fungal communities, the α-diversity indices and community compositions of the functional guilds (e.g., ECM and saprotrophic) differed significantly among the seven plantations (Supplementary Tables S2, S3). In particular, the mixed plantation of *P. koraiensis* and *F. mandshurica* showed higher ECM fungal diversity, whereas that of *P.* *koraiensis* and *J. mandshurica* showed higher saprophytic fungal diversity. We performed RDA to reveal potential relationships between functional genera and soil nutrients and found that saprotrophic fungi were most closely associated with higher soil nutrient levels, which might be attributable to their functional traits associated with the decomposition and nutrient cycling of leaf litter in soil (Ceci et al., 2018). However, ECM fungi were mainly distributed in low-nutrient areas. Marty et al. (2019) proposed that ECM fungi, as nutrient conduits, satisfy the need of host trees, whereas the role of saprotrophs is mainly to maintain nutritional homeostasis of the ecosystem. Furthermore, soils with a higher abundance of ECM fungi showed lower pH. Craig et al. (2018) reported that soils abundant in ECM fungi tended to be more acidic and that a low soil pH prevented the recession in ECM systems while influencing other soil microbial communities. Other studies that saprotrophic fungal biomass increased as the abundance of ECM fungal biomass declined (Fernandez and Kennedy, 2016). However, ECM fungi can exist in a state of competition with saprotrophic fungi by forming recalcitrant tissues to retard decomposition rates or inhibit the activities of saprotrophic fungi (Fernandez et al., 2013; Fernandez and Kennedy, 2016; Craig et al., 2018).

Network analysis can help identify interactions and provide a holistic view of microbial ecosystems to allow a better understanding of interactions within functional fungal communities (Banerjee et al., 2016). In this study, co-occurring network analysis suggested positive associations between functional genera, with significant positive associations among 45 OTUs (nodes) from 33 genera (*p*< 0.05; Figure 3). The resulting nodes were mainly divided into two networks, one of which comprised saprophytic fungi with 25 nodes, wherein *Mortierella* showed a high degree of connections with other genera and a higher relative abundance. The keystone species (high-degree species) in a co-occurrence network of soil microorganisms could have a large impact on the community composition (Berry and Widder, 2014; Chao et al., 2016). In the present study, the resulting network suggested that *Mortierella*, which is capable of transforming phosphorus from an insoluble to a soluble form that can then be directly utilized by plants, played a central role, with a significant impact on many other soil microbes, in both pure and mixed plantations (Osorio and Habte, 2013). The second network comprised ECM fungi with 18 nodes. ECM fungi provide multiple services to plants and ecosystems through their ability to convert and provide soil nutrients to plants (Liu Y.B. et al., 2018). Here, we found that *Tomentella* displayed a high degree of connections with other genera, followed by *Amphinema*, *Clavulina*, and *Russula*. Previous reports concerning the genus *Tomentella* demonstrated that *Tomentella* spp. were most frequently associated with ECM fungi in temperate forests (Jakucs et al., 2005) and were among the richest genera in a diverse ECM fungal community from *Abies religiosa* (also including *Inocybe*, *Russula*, and *Clavulina*) (Arguelles-Moyao et al., 2017). Moreover, the 91 interactions found in the network implied that close synergistic relationships could be formed within functional genera (e.g., ECM and saprotrophs) through direct or indirect interactions. Potential synergistic partnerships between functional genera in co-occurring networks would tend to strengthen the conversion of nutrients to promote ecological functions in a forest. Although ecological functions of soil microbial communities can influence plant development (Harris, 2009), selective actions of plants can also regulate the species composition and activity of soil microorganisms (Hartmann et al., 2009). It would be advantageous for plant species to broaden the niche and adapt to new environments by expanding the patterns and preferences of nutrient uptake with the help of symbiotic microbes (Wu et al., 2013; Afkhami et al., 2014). Thus, these results will facilitate effective forest management.

## Conclusion

Our study assessed the influence of mixed conifer broadleaf plantations in typical temperate forests on soil characteristics and the composition of functional fungal communities. We found that soil fungal communities responded differently to changes associated with soil nutrient levels, indicating the existence of interdependent regulatory mechanisms between different forest types, soil nutrients, and soil microbial communities. Our results indicated higher saprotrophic fungal richness and diversity and soil nutrient levels in a mixed forest of *P. koraiensis* and *J. mandshurica* compared with those in a mixed forest of *P. koraiensis* and *F. mandshurica*, which showed a lower level of soil nutrients and higher abundance of ECM fungi. A positive association between soil nutrients and soil fungi enhanced the ability of the mixed forests to withstand changes in the external environment. This study improves our understanding of the advantages of mixed forests (especially with ECM-associated tree species) by revealing the relationship between soil nutrients and functional fungal diversity.

## Author Contributions

GS contributed to conceiving and designing the experiments. DW, MZ, and WL performed the experiments and the data analysis. DW, MP, and XS drew the figures and wrote sections of the manuscript.

## Conflict of Interest Statement

The authors declare that the research was conducted in the absence of any commercial or financial relationships that could be construed as a potential conflict of interest.

## Footnotes

1 http://drive5.com/uparse/

## References

[B1] Adams R. I. Miletto M. Taylor J. W. Bruns T. D. (2013). Dispersal in microbes: fungi in indoor air are dominated by outdoor air and show dispersal limitation at short distances. *ISME J.* 7 1262–1273. 10.1038/ismej.2013.28 23426013 PMC3695294

[B2] Afkhami M. E. McIntyre P. J. Strauss S. Y. (2014). Mutualist-mediated effects on species’ range limits across large geographic scales. *Ecol. Lett.* 17 1265–1273. 10.1111/ele.12332 25052023

[B3] Arguelles-Moyao A. Garibay-Orijel R. Marquez-Valdelamar L. M. Arellano-Torres E. (2017). Clavulina-membranomyces is the most important lineage within the highly diverse ectomycorrhizal fungal community of Abies religiosa. *Mycorrhiza* 27 53–65. 10.1007/s00572-016-0724-1 27562509

[B4] Banerjee S. Kirkby C. A. Schmutter D. Bissett A. Kirkegaard J. A. Richardson A. E. (2016). Network analysis reveals functional redundancy and keystone taxa amongst bacterial and fungal communities during organic matter decomposition in an arable soil. *Soil Biol. Biochem.* 97 188–198. 10.1016/j.soilbio.2016.03.017

[B5] Becquer A. Garcia K. Amenc L. Rivard C. Dore J. Trives-Segura C. (2018). The hebeloma cylindrosporum HcPT2 Pi transporter plays a key role in ectomycorrhizal symbiosis. *New Phytol.* 220 1185–1199. 10.1111/nph.15281 29944179

[B6] Berry D. Widder S. (2014). Deciphering microbial interactions and detecting keystone species with co-occurrence networks. *Front. Microbiol.* 5:219. 10.3389/fmicb.2014.00219 24904535 PMC4033041

[B7] Blagodatskaya Å. Kuzyakov Y. (2008). Mechanisms of real and apparent priming effects and their dependence on soil microbial biomass and community structure: critical review. *Biol. Fertil. Soils* 45 115–131. 10.1007/s00374-008-0334-y

[B8] Boberg J. B. Ihrmark K. Lindahl B. D. (2011). Decomposing capacity of fungi commonly detected in Pinus sylvestris needle litter. *Fungal Ecol.* 4 110–114. 10.1016/j.funeco.2010.09.002

[B9] Buee M. Reich M. Murat C. Morin E. Nilsson R. H. Uroz S. (2009). 454 pyrosequencing analyses of forest soils reveal an unexpectedly high fungal diversity. *New Phytol.* 184 449–456. 10.1111/j.1469-8137.2009.03003.x 19703112

[B10] Busse M. D. Beattie S. E. Powers R. F. Sanchez F. G. Tiarks A. E. (2006). Microbial community responses in forest mineral soil to compaction, organic matter removal, and vegetation control. *Can. J. Forest Res.* 36 577–588. 10.1139/X05-294

[B11] Cannell M. G. R. Malcolm D. C. Robertson P. A. (1992). The Ecology of Mixed-Species Stands of Trees. Hoboken, NJ: Blackwell Scientific.

[B12] Caporaso J. G. Kuczynski J. Stombaugh J. Bittinger K. Bushman F. D. Costello E. K. (2010). QIIME allows analysis of high-throughput community sequencing data. *Nat. Methods* 7 335–336. 10.1038/nmeth.f.303 20383131 PMC3156573

[B13] Ceci A. Pinzari F. Russo F. Persiani A. M. Gadd G. M. (2018). Roles of saprotrophic fungi in biodegradation or transformation of organic and inorganic pollutants in co-contaminated sites. *Appl. Microbiol. Biotechnol.* 103 53–68. 10.1007/s00253-018-9451-1 30362074

[B14] Chao Y. Q. Liu W. S. Chen Y. M. Chen W. H. Zhao L. H. Ding Q. B. (2016). Structure, variation, and co-occurrence of soil microbial communities in abandoned sites of a rare earth elements mine. *Environ. Sci. Technol.* 50 11481–11490. 10.1021/acs.est.6b02284 27670106

[B15] Chauvat M. Titsch D. Zaytsev A. S. Wolters V. (2011). Changes in soil faunal assemblages during conversion from pure to mixed forest stands. *Forest Ecol Manag.* 262 317–324. 10.1016/j.foreco.2011.03.037

[B16] Chen T. H. Chiu C. Y. Tian G. (2005). Seasonal dynamics of soil microbial biomass in coastal sand dune forest. *Pedobiologia* 49 645–653. 10.1016/j.pedobi.2005.06.005

[B17] Clasen B. E. Silveira A. D. Baldoni D. B. Montagner D. F. Jacques R. J. S. Antoniolli Z. I. (2018). Characterization of Ectomycorrhizal species through molecular biology tools and morphotyping. *Sci. Agric.* 75 246–254. 10.1590/1678-992X-2016-0419

[B18] Comerford N. B. Franzluebbers A. J. Stromberger M. E. Morris L. Markewitz D. Moore R. (2013). Assessment and evaluation of soil ecosystem services. *Soil Horiz.* 54 1–14.

[B19] Courty P. E. Buee M. Diedhiou A. G. Frey-Klett P. Le Tacon F. Rineau F. (2010). The role of ectomycorrhizal communities in forest ecosystem processes: new perspectives and emerging concepts. *Soil Biol. Biochem.* 42 679–698. 10.1016/j.soilbio.2009.12.006

[B20] Craig M. E. Turner B. L. Liang C. Clay K. Johnson D. J. Phillips R. P. (2018). Tree mycorrhizal type predicts within-site variability in the storage and distribution of soil organic matter. *Glob. Change Biol.* 24 3317–3330. 10.1111/gcb.14132 29573504

[B21] Cromack K. Sollins P. Todd R. L. Crossley D. A. Fender W. M. Fogel R. (1977). *Soil Microorganism—Arthropod Interactions: Fungi as Major Calcium and Sodium Sources.* Berlin: Springer

[B22] Detheridge A. P. Comont D. Callaghan T. M. Bussell J. Brand G. Gwynn-Jones D. (2018). Vegetation and edaphic factors influence rapid establishment of distinct fungal communities on former coal-spoil sites. *Fungal Ecol.* 33 92–103. 10.1016/j.funeco.2018.02.002

[B23] Dong W. Y. Zhang X. Y. Dai X. Q. Fu X. L. Yang F. T. Liu X. Y. (2014). Changes in soil microbial community composition in response to fertilization of paddy soils in subtropical China. *Appl. Soil Ecol.* 84 140–147. 10.1016/j.apsoil.2014.06.007

[B24] Evans J. (1992). *Plantation Forestry in the Tropics: Tree Planting for Industrial, Social, Environmental, and Agroforestry Purposes.* Oxford: Oxford University Press.

[B25] Fernandez C. W. Kennedy P. G. (2016). Revisiting the ’Gadgil effect’: do interguild fungal interactions control carbon cycling in forest soils? *New Phytol.* 209 1382–1394. 10.1111/nph.13648 26365785

[B26] Fernandez C. W. McCormack M. L. Hill J. M. Pritchard S. G. Koide R. T. (2013). On the persistence of Cenococcum geophilum ectomycorrhizas and its implications for forest carbon and nutrient cycles. *Soil Biol. Biochem.* 65 141–143. 10.1016/j.soilbio.2013.05.022

[B27] Flores-Rentería D. Rincón A. Valladares F. Yuste J. (2016). Agricultural matrix affects differently the alpha and beta structural and functional diversity of soil microbial communities in a fragmented mediterranean holm oak forest. *Soil Biol. Biochem.* 92 79–90. 10.1016/j.soilbio.2015.09.015

[B28] Forrester D. I. (2014). The spatial and temporal dynamics of species interactions in mixed-species forests: from pattern to process. *Forest Ecol. Manag.* 312 282–292. 10.1016/j.foreco.2013.10.003

[B29] Gessner M. O. Swan C. M. Dang C. K. Mckie B. G. Bardgett R. D. Wall D. H. (2010). Diversity meets decomposition. *Trends Ecol. Evol.* 25 372–380. 10.1016/j.tree.2010.01.010 20189677

[B30] Gu J. C. Chu X. Wang Z. Q. (2010). Diameter growth of fraxinus mandshurica and its relation to silviculture techniques. *J. Northeast For. Univ.* 38 7–9.

[B31] Gusman J. K. Lin C. Y. Shih Y. C. (2014). The optimum submerged culture condition of the culinary-medicinal white jelly mushroom (*Tremellomycetes*) and Its antioxidant properties. *Int. J. Med. Mushrooms* 16 293–302. 10.1615/IntJMedMushr.v16.i3.90 24941170

[B32] Harris J. (2009). Soil microbial communities and restoration ecology: facilitators or followers? *Science* 325 573–574. 10.1126/science.1172975 19644111

[B33] Hartmann A. Schmid M. van Tuinen D. Berg G. (2009). Plant driven selection of microbes. *Plant Soil* 321 235–257. 10.1007/s11104-008-9814-y

[B34] Ingham R. E. Trofymow J. A. Ingham E. R. Coleman D. C. (1985). Interactions of bacteria, fungi, and their nematode grazers: effects on nutrient cycling and plant growth. *Ecol monogr.* 55 119–140. 10.2307/1942528

[B35] ISO 15685 (2012). *Soil Quality - Determination of Potential Nitrification and Inhibition of Nitrification -Rapid test by Ammonium Oxidation.* Rome: ISO

[B36] Jakucs E. Kovacs G. M. Agerer R. Romsics C. Eros-Honti Z. (2005). Morphological-anatomical characterization and molecular identification of *Tomentella stuposa* ectomycorrhizae and related anatomotypes. *Mycorrhiza* 15 247–258. 10.1007/s00572-004-0326-1 15517422

[B37] Jiang Y. Chen C. Xu Z. Liu Y. (2012). Effects of single and mixed species forest ecosystems on diversity and function of soil microbial community in subtropical China. *J. Soils Sediments* 12 228–240. 10.1007/s11368-011-0442-4

[B38] Karst J. Burns C. Cale J. A. Antunes P. M. Woods M. Lamit L. J. (2018). Tree species with limited geographical ranges show extreme responses to ectomycorrhizas. *Glob. Ecol. Biogeogr.* 27 839–848. 10.1111/geb.12745

[B39] Khan S. A. Mulvaney R. L. Hoeft R. G. (2001). A simple soil test for detecting sites that are nonresponsive to nitrogen fertilization. *Soil Sci. Soc. Am. J.* 65 1751–1760. 10.2136/sssaj2001.1751

[B40] Li C. C. Li Q. R. Qiao N. Xu X. L. Li Q. K. Wang H. M. (2016). Inorganic and organic nitrogen uptake by nine dominant subtropical tree species. *Iforest* 9 253–258. 10.3832/ifor1502-008

[B41] Li H. L. Ostermann A. Karunarathna S. C. Xu J. C. Hyde K. D. Mortimer P. E. (2018). The importance of plot size and the number of sampling seasons on capturing macrofungal species richness. *Fungal Biol.* 122 692–700. 10.1016/j.funbio.2018.03.004 29880204

[B42] Liu M. Mao Z. J. Li Y. Sun T. Li X. H. Huang W. (2016). Response of radial growth of pinus koraiensis in broad-leaved korean pine forests with different latitudes to climatical factors. *J. Appl. Ecol.* 27 1341–1352. 10.13287/j.1001-9332.201605.020 29732793

[B43] Liu Y. B. Chen H. M. Mou P. (2018). Spatial patterns nitrogen transfer models of ectomycorrhizal networks in a mongolian scotch pine plantation. *J For. Res.* 29 339–346. 10.1007/s11676-017-0454-z

[B44] Liu Y. P. Sun Q. B. Li J. Lian B. (2018). Bacterial diversity among the fruit bodies of ectomycorrhizal and saprophytic fungi and their corresponding hyphosphere soils. *Sci. Rep.* 8:11672. 10.1038/s41598-018-30120-6 30076360 PMC6076286

[B45] Lu R. K. (2000). *Analytical Methods of Soil and Agricultural Chemistry.* Beijing: China Agricultural Science and Technology Press

[B46] Luo S. Schmid B. De Deyn G. B. Yu S. X. (2018). Soil microbes promote complementarity effects among co-existing trees through soil nitrogen partitioning. *Funct. Ecol.* 32 1879–1889. 10.1111/1365-2435.13109

[B47] Ma J. C. Ibekwe A. M. Yang C. H. Crowley D. E. (2016). Bacterial diversity and composition in major fresh produce growing soils affected by physiochemical properties and geographic locations. *Sci. Total Environ.* 563–564, 199–209. 10.1016/j.scitotenv.2016.04.122 27135583

[B48] Marty K. J. Rachael H. D. Hawkins B. J. (2019). Saprotrophic and ectomycorrhizal fungal sporocarp stoichiometry (C: N: P) across temperate rainforests as evidence of shared nutrient constraints among symbionts. *New Phytol.* 221 482–492. 10.1111/nph.15380 30084239

[B49] Millanes A. M. Diederich P. Ekman S. Wedin M. (2011). Phylogeny and character evolution in the jelly fungi (*Tremellomycetes, Basidiomycota, Fungi*). *Mol. Phylogenet. Evol.* 61 12–28. 10.1016/j.ympev.2011.05.014 21664282

[B50] Nagati M. Roy M. Manzi S. Richard F. Desrochers A. Gardes M. (2018). Impact of local forest composition on soil fungal communities in a mixed boreal forest. *Plant Soil* 432 345–357. 10.1007/s11104-018-3806-3

[B51] Nguyen N. H. Song Z. Bates S. T. Branco S. Tedersoo L. Menke J. (2016). FUNGuild: an open annotation tool for parsing fungal community datasets by ecological guild. *Fungal Ecol.* 20 241–248. 10.1016/j.funeco.2015.06.006

[B52] Nygren C. M. R. Edqvist J. Elfstrand M. Heller G. Taylor A. F. S. (2007). Detection of extracellular protease activity in different species and genera of ectomycorrhizal fungi. *Mycorrhiza* 17 241–248. 10.1007/s00572-006-0100-107 17216502

[B53] Olsen S.R. Sommers L.E. (1982). “Phosphorus,” in Page A.L. Miller R.H. Keeney D.R (eds.) *Methods of Soil Analysis. 2nd Edn*, (Madison, WI: Soil Science Society of America, Inc), 403–430.

[B54] Osorio N. W. Habte M. (2013). Synergistic effect of a phosphate-solubilizing fungus and an arbuscular mycorrhizal fungus on leucaena seedlings in an Oxisol fertilized with rock phosphate. *Botany* 91 274–281. 10.1139/cjb-2012-0226

[B55] Paquette A. Messier C. (2010). The role of plantations in managing the world’s forests in the Anthropocene. *Front. Ecol. Environ.* 8 27–34. 10.1890/080116

[B56] Parks D. H. Tyson G. W. Hugenholtz P. Beiko R. G. (2014). STAMP: statistical analysis of taxonomic and functional profiles. *Bioinformatics* 30 3123–3124. 10.1093/bioinformatics/btu494 25061070 PMC4609014

[B57] Peay K. G. (2016). The mutualistic niche: mycorrhizal symbiosis and community dynamics. *Annu. Rev. Ecol. Evol. Syst.* 47 143–164. 10.1146/annurev-ecolsys-121415-032100

[B58] Powlson D. Prookes P. Christensen B. (1987). Measurement of soil microbial biomass provides an early indication of changes in total soil organic matter due to straw incorporation. *Soil biol. Biochem.* 19 159–164. 10.1016/0038-0717(87)90076-9

[B59] Priha O. Smolander A. (1997). Microbial biomass and activity in soil and litter under Pinus sylvestris, Picea abies and Betula pendula at originally similar field afforestation sites. *Biol. Fertil. Soils* 24 45–51. 10.1007/BF01420219

[B60] Reeves D. W. (1997). The role of soil organic matter in maintaining soil quality in continuous cropping systems. *Soil Tillage Res.* 43 131–167. 10.1016/S0167-1987(97)00038-X

[B61] Richter A. Schöning I. Kahl T. Bauhus J. Ruess L. (2018). Regional environmental conditions shape microbial community structure stronger than local forest management intensity. *For. Ecol. Manag.* 409 250–259. 10.1016/j.foreco.2017.11.027

[B62] Saetre P. Bååth E. (2009). Spatial variation and patterns of soil microbial community structure in a mixed spruce-birch stand. *Soil Biol. Biochem.* 32 909–917. 10.1016/S0038-0717(99)00215-1

[B63] Seidel D. Leuschner C. Scherber C. Beyer F. Wommelsdorf T. Cashman M. J. (2013). The relationship between tree species richness, canopy space exploration and productivity in a temperate broad-leaf mixed forest. *For. Ecol. Manag.* 310 366–374. 10.1016/j.foreco.2013.08.058

[B64] Shade A. Handelsman J. (2012). Beyond the venndiagram: the hunt for a core microbiome. *Environ. Microbiol.* 14 4–12. 10.1111/j.1462-2920.2011.02585.x 22004523

[B65] Shannon P. Markiel A. Ozier O. Baliga N. S. Wang J. T. Ramage D. (2003). Cytoscape: a software environment for integrated models of biomolecular interaction networks. *Genome Res.* 13 2498–2504. 10.1101/gr.1239303 14597658 PMC403769

[B66] Shen W. S. Ni Y. Y. Gao N. Bian B. Y. Zheng S. N. Lin X. G. (2016). Bacterial community composition is shaped by soil secondary salinization and acidification brought on by high nitrogen fertilization rates. *Appl. Soil Ecol.* 108 76–83. 10.1016/j.apsoil.2016.08.005

[B67] Simard S. W. Beiler K. J. Bingham M. A. Deslippe J. R. Philip L. J. Teste F. P. (2012). Mycorrhizal networks: mechanisms, ecology and modelling. *Fungal Biol. Rev.* 26 39–60. 10.1016/j.fbr.2012.01.001

[B68] Singh K. Singh B. Singh R. R. (2012). Changes in physico-chemical, microbial and enzymatic activities during restoration of degraded sodic land: ecological suitability of mixed forest over monoculture plantation. *Catena* 96 57–67. 10.1016/j.catena.2012.04.007.

[B69] Song Z. Vail A. Sadowsky M. J. Schilling J. S. (2015). Influence of Hyphal Inoculum potential on the competitive success of fungi colonizing wood. *Microb. Ecol.* 69 758–767. 10.1007/s00248-015-0588-5 25750000

[B70] Sun N. Liu Q. (2015). Adjusting the tree species composition of hardwood broad-leaved mixed forests of Fraxinus mandshurica, Juglandis mandshuricae and Phyllostachys sulphurea. *J. Northwest For. Univ.* 30 120–125. 10.3969/j.issn.1001-7461.2015.01.19

[B71] Talbot J. M. Bruns T. D. Smith D. P. Branco S. Glassman S. I. Erlandson S. (2013). Independent roles of ectomycorrhizal and saprotrophic communities in soil organic matter decomposition. *Soil Biol. Biochem.* 57 282–291. 10.1016/j.soilbio.2012.10.004

[B72] Teste F. P. Veneklaas E. J. Dixon K. W. Lambers H. (2014). Complementary plant nutrient-acquisition strategies promote growth of neighbour species. *Funct. Ecol.* 28 819–828. 10.1111/1365-2435.12270

[B73] Turnbaugh P. J. Ley R. E. Hamady M. Fraser-Liggett C. M. Knight R. Gordon J. I. (2007). The human microbiome project: exploring the microbial part of ourselves in a changing world. *Nature* 449 804–810. 10.1038/nature06244 17943116 PMC3709439

[B74] Ushio M. Kitayama K. Balser T. C. (2010). Tree species effects on soil enzyme activities through effects on soil physicochemical and microbial properties in a tropical montane forest on Mt. *Kinabalu, Borneo*. *Pedobiologia* 53 227–233. 10.1016/j.pedobi.2009.12.003

[B75] Van Den Wollenberg A. L. (1977). Redundancy analysis an alternative for canonical correlation analysis. *Psychometrika* 42 207–219. 10.1007/BF02294050

[B76] Van Der Heijden M. G. Bardgett R. D. Van Straalen N. M. (2008). The unseen majority: soil microbes as drivers of plant diversity and productivity in terrestrial ecosystems. *Ecol. Lett.* 11 296–310. 10.1111/j.1461-0248.2007.01139.x 18047587

[B77] Wang B. Y. Zhao X. Y. Wang H. W. Jiang G. Y. Shen G. Wang L. K. (2019). Variance analysis of growth characteristics of pinus koraiensis Half-sib families. *J. Northeast For. Univ.* 47 8–11. 10.13759/j.cnki.dlxb.2019.04.002

[B78] Wang H. Liu S. Wang J. Shi Z. Lu L. Zeng J. (2013). Effects of tree species mixture on soil organic carbon stocks and greenhouse gas fluxes in subtropical plantations in China. *For. Ecol. Manag.* 300 4–13. 10.1016/j.foreco.2012.04.005

[B79] Wang W. X. Shi Z. M. Luo D. Liu S. R. Lu L. H. (2013). Characteristics of soil microbial biomass and community composition in three types of plantations in southern subtropical area of China. *J. Appl. Ecol.* 24 1784–1792. 24175505

[B80] Weber C. F. Lockhart J. S. Charaska E. Aho K. Lohse K. A. (2014). Bacterial composition of soils in ponderosa pine and mixed conifer forests exposed to different wildfire burn severity. *Soil Biol. Biochem.* 69 242–250. 10.1016/j.soilbio.2013.11.010

[B81] White C. Tardif J. C. Adkins A. Staniforth R. (2005). Functional diversity of microbial communities in the mixed boreal plain forest of central Canada. *Soil Biol. Biochem.* 37 1359–1372. 10.1016/j.soilbio.2004.12.007

[B82] Wilhelm R. C. Cardenas E. Leung H. Szeitz A. Jensen L. D. Mohn W. W. (2017). Long-Term enrichment of stress-tolerant cellulolytic soil populations following timber harvesting evidenced by multi-omic stable isotope probing. *Front. Microbiol.* 8:537 10.3389/fmicb.2017.00537 PMC5386986 28443069

[B83] Wu J. R. Ma H. C. Xu X. L. Qiao N. Guo S. T. Liu F. (2013). Mycorrhizas alter nitrogen acquisition by the terrestrial orchid *Cymbidium goeringii*. *Ann Bot. London* 111 1181–1187. 10.1093/aob/mct062 23532045 PMC3662508

[B84] Xiao C. C. Ran S. J. Huang Z. W. Liang J. P. (2016). Bacterial Diversity and Community Structure of Supragingival Plaques in Adults with Dental Health or Caries Revealed by 16S Pyrosequencing. *Front. Microbiol.* 7:1145 10.3389/fmicb.2016.01145 PMC4956651 27499752

[B85] Xiong W. Li Z. Liu H. Xue C. Zhang R. Wu H. (2015). The effect of long-term continuous cropping of black pepper on soil bacterial communities as determined by 454 pyrosequencing. *PloS One* 10:e0136946. 10.1371/journal.pone.0136946 26317364 PMC4552827

[B86] Xu F. Cai T. J. Yang X. Sui W. Z. (2017). Soil fungal community variation by large-scale reclamation in Sanjiang plain, China. *Ann. Microb.* 67 679–689. 10.1007/s13213-017-1296-9

[B87] Zhang N. L. Li Y. N. Wubet T. Bruelheide H. Liang Y. Purahong W. (2018). Tree species richness and fungi in freshly fallen leaf litter: unique patterns of fungal species composition and their implications for enzymatic decomposition. *Soil Biol. Biochem.* 127 120–126. 10.1016/j.soilbio.2018.09.023

[B88] Žifčáková L. Větrovský T. Howe A. Baldrian P. (2016). Microbial activity in forest soil reflects the changes in ecosystem properties between summer and winter. *Environ. Microbiol.* 18 288–301. 10.1111/1462-2920.13026 26286355

