# Supplementary material for: Variations in Soil Functional Fungal Community Structure Associated With Pure and Mixed Plantations in Typical Temperate Forests of China
Source: Front Microbiol. 2019 Jul 16;10:1636. doi: 10.3389/fmicb.2019.01636 (PMC6646410; doi:10.3389/fmicb.2019.01636)
Supplement: Supplementary file 1 [file Data_Sheet_1.PDF]

SUPPLEMENTARY MATERIAL

Variations in soil functional fungal-community structure associated with pure and mixed forests in typical temperate forests of China

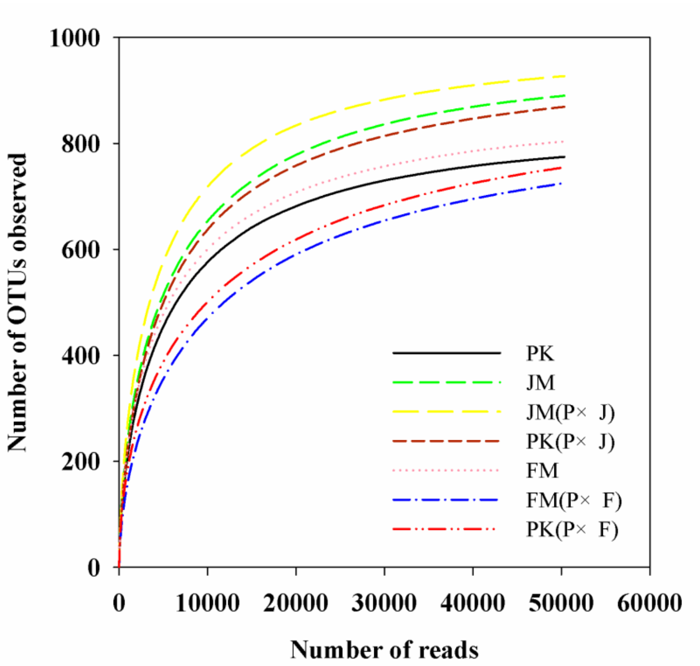

Supplementary Figure S1 Rarefaction curves depicting the effect of ITS sequence number on the number of OTUs (mean value).

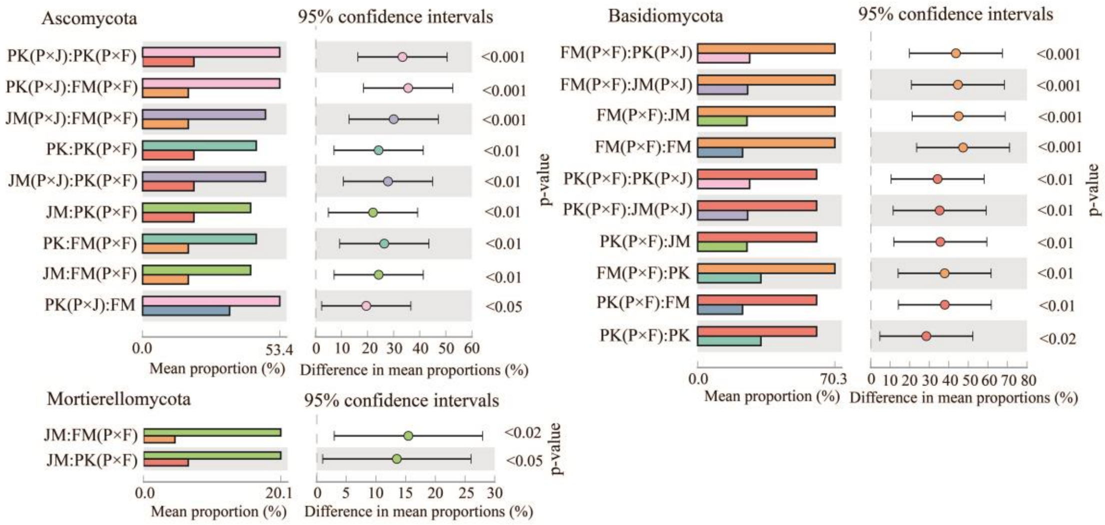

Supplementary Figure S2 The significance test of the relative abundance of the dominant phyla from different plantations.

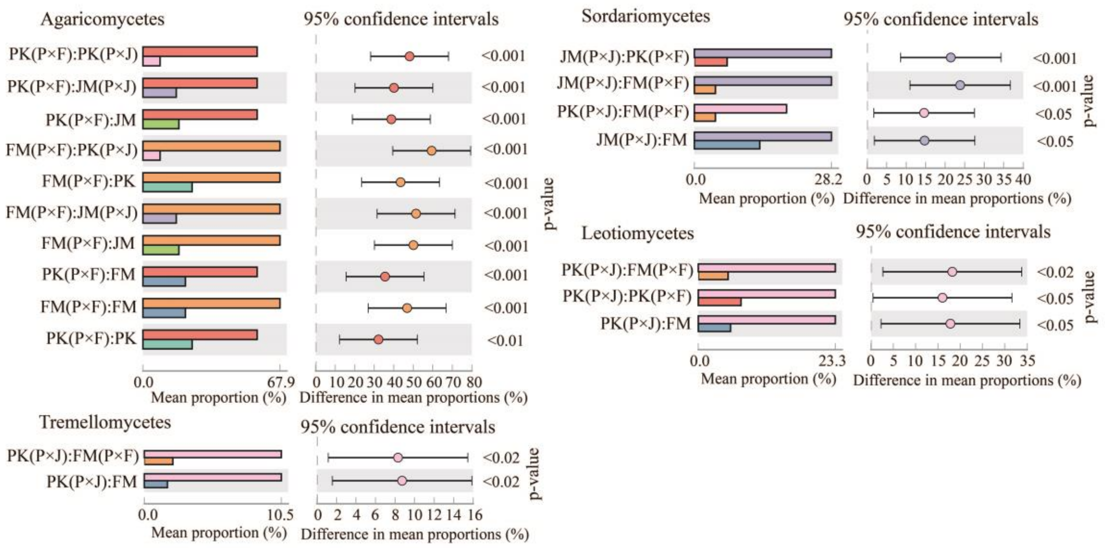

Supplementary Figure S3 The significance test of the relative abundance of the dominant classes from different plantations.

Supplementary Table 1 The read numbers of dominant OTUs from different plantations (Top 20 OTUs).

	PK	JM	JM(P×J)	PK(P×J)	FM	FM(P×F)	PK(P×F)	*F*	*P*
OTU1454	6126±4143bc	142±52a	1094±403a	6984±5164c	0a	1244±120a	1801±629ab	3.87	0.017
OTU1949	3183±416c	5491±121d	2382±21bc	1689±322ab	1735±825ab	929±661a	1467±941ab	21.58	<0.001
OTU1451	7±2a	161±82a	144±56a	43±15a	7880±4863b	790±165a	65±12a	7.53	0.001
OTU3158	1162±369a	1073±615a	840±320a	3577±2141b	70±34a	532±106a	1125±408a	4.90	0.007
OTU3547	2640±721b	2±2a	0a	4±4a	0a	1±1a	5649±1846c	25.87	<0.001
OTU1144	0a	1±1a	0a	0a	0a	7324±4330b	0.3±0.6a	8.58	<0.001
OTU19	1171±608	1682±1377	1634±1241	939±586	883±202	223±46	228±89	1.75	0.181
OTU1421	451±73c	0a	32±9a	161±60b	0a	4364±155f	1699±37d	0.002	<0.001
OTU2232	45±23a	134±80a	3±5a	0a	2911±2526b	0a	3476±2474b	4.03	0.015
OTU3762	208±58a	384±205a	4947±3882b	433±185a	256±46a	32±12a	310±74a	4.35	0.11
OTU2963	1646±878b	891±522ab	1087±486ab	1064±598ab	863±623ab	193±166a	382±323a	2.24	0.1
OTU1432	1175±197b	938±240b	1671±230c	971±73b	853±329b	195±153a	248±167a	17.73	<0.001
OTU394	0a	0a	0a	8±7a	21±29a	5260±3027b	23±4a	9.03	<0.001
OTU2161	1214±642b	9±5a	379±238ab	2553±1455c	0a	172±127ab	436±322ab	6.62	0.002
OTU679	190±42a	51±34a	195±38a	3471±1731b	3±5a	33±21a	298±94a	11.22	<0.001
OTU2716	0a	0a	0a	0a	0a	3816±1170b	350±40a	31.15	<0.001
OTU1956	472±304	482±251	1000±477	661±416	941±719	249±211	236±202	1.70	0.194
OTU3597	643±27c	1012±440d	619±144bc	592±61bc	710±139cd	149±92a	268±132ab	6.52	0.002
OTU2601	8±2a	4±6a	58±20a	7±3a	3±4a	0a	3616±95b	0.004	<0.001
OTU1379	538±135b	0a	211±39ab	71±44ab	0a	2414±653c	436±140ab	33.21	<0.001

Note: The different letters indicate a significant difference among the seven treatments, Duncan's multiple range test.

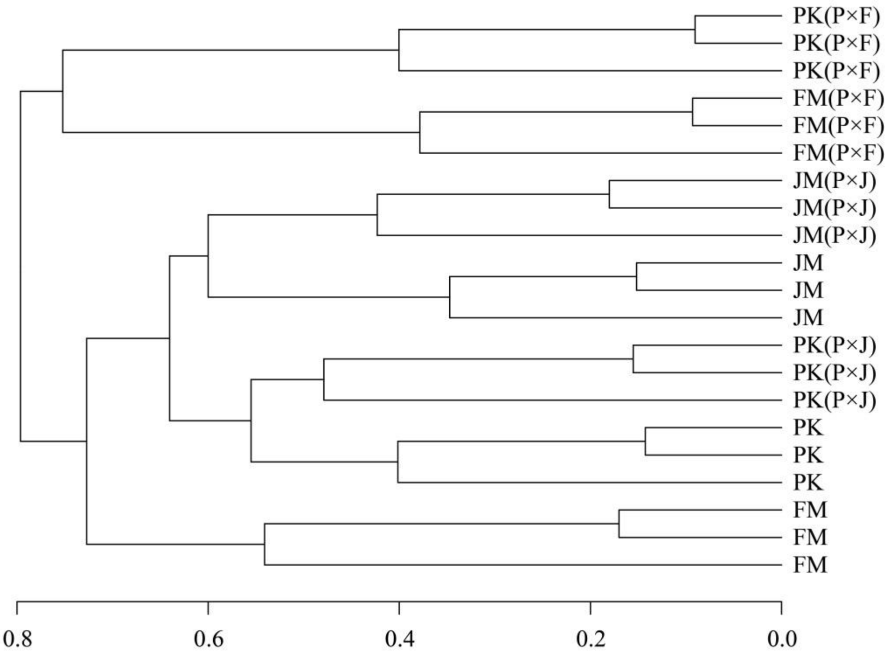

Supplementary Figure S4 Cluster analysis of soil samplesat the OTU level from different plantations.

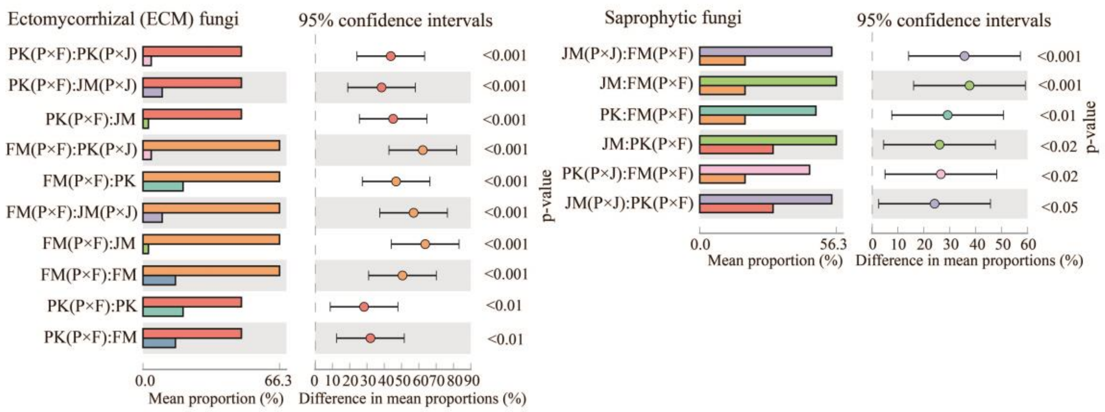

Supplementary Figure S5 The significance test of the total abundance of ECM and saprotrophic fungi from different plantations.

Supplementary Table 2 Richness and diversity estimators of ECM fungal community in the different types of plantation.

Sample	OTU		ACE		Chao		Shannon	
	Mean	SE	Mean	SE	Mean	SE	Mean	SE
PK	79ab	3.5	81b	2.00	82a	1.7	2.69ab	0.13
JM	66a	7.9	69ab	6.7	69a	6.4	2.71ab	0.72
JM(P×J)	69a	2.1	72ab	2.7	82a	20.5	2.06a	0.07
PK(P×J)	63a	11.7	66a	8.1	67a	8.6	2.69ab	0.22
FM	72a	7.2	74ab	5.8	74a	6.1	1.98a	0.56

FM(P×F)	102b	14.8	108c	15.0	110b	17.6	2.63ab	0.32
PK(P×F)	136c	7.5	144d	4.6	150c	0.6	3.09b	0.19
F	26.804^{***}		42.572^{***}		20.926^{***}		3.110^{*}	
P	<0.001		<0.001		<0.001		0.038	

Note: The different letters indicate a significant difference among the seven treatments, Duncan's multiple range test.

Supplementary Table 3 Richness and diversity estimators of saprotrophic fungal community in the different types of plantation.

Sample	OTU		ACE		Chao		Shannon	
	Mean	SE	Mean	SE	Mean	SE	Mean	SE
PK	281abc	7.8	289a	12.2	290a	14.0	3.85a	0.17
JM	301abc	18.0	311ab	18.3	311abc	20.1	3.74a	0.21
JM(P×J)	338c	20.5	347c	18.5	352c	20.2	3.97a	0.55
PK(P×J)	331bc	36.5	344c	25.5	349bc	17.8	3.82a	0.47
FM	288abc	29.9	299a	16.1	304ab	8.1	4.21a	0.30
FM(P×F)	264a	50.5	295a	26.5	296a	25.5	3.67a	0.58
PK(P×F)	269ab	48.2	299a	38.1	303ab	50.2	3.46a	0.46
F	2.278		3.042^{*}		2.875^{*}		0.959	
P	0.096		0.040		0.049		0.486	

Note: The different letters indicate a significant difference among the seven treatments, Duncan's multiple range test.
